# TGF-β1 autocrine signalling and enamel matrix components

**DOI:** 10.1038/srep33644

**Published:** 2016-09-16

**Authors:** Saeko Kobayashi-Kinoshita, Yasuo Yamakoshi, Kazuo Onuma, Ryuji Yamamoto, Yoshinobu Asada

**Affiliations:** 1 Department of Pediatric Dentistry, School of Dental Medicine, Tsurumi University, 2-1-3 Tsurumi, Tsurumi-ku, Yokohama 230-8501, Japan; 2 Department of Biochemistry and Molecular Biology, School of Dental Medicine, Tsurumi University, 2-1-3 Tsurumi, Tsurumi-ku, Yokohama 230-8501, Japan; 3 National Institute of Advanced Industrial Science & Technology, Central 6, 1–1-1 Higashi, Tsukuba, Ibaraki 305-8566, Japan

## Abstract

Transforming growth factor-β1 (TGF-β1) is present in porcine enamel extracts and is critical for proper mineralization of tooth enamel. Here, we show that the mRNA of latent TGF-β1 is expressed throughout amelogenesis. Latent TGF-β1 is activated by matrix metalloproteinase 20 (MMP20), coinciding with amelogenin processing by the same proteinase. Activated TGF-β1 binds to the major amelogenin cleavage products, particularly the neutral-soluble P103 amelogenin, to maintain its activity. The P103 amelogenin-TGF-β1 complex binds to TGFBR1 to induce TGF-β1 signalling. The P103 amelogenin-TGF-β1 complex is slowly cleaved by kallikrein 4 (KLK4), which is secreted into the transition- and maturation-stage enamel matrix, thereby reducing TGF-β1 activity. To exert the multiple biological functions of TGF-β1 for amelogenesis, we propose that TGF-β1 is activated or inactivated by MMP20 or KLK4 and that the amelogenin cleavage product is necessary for the in-solution mobility of TGF-β1, which is necessary for binding to its receptor on ameloblasts and retention of its activity.

Enamel formation progresses in three stages: secretory, transition and maturation. Enamel proteins are abundant during the secretory stage and are slowly processed by matrix metalloproteinase 20 (MMP20), which is expressed by secretory-stage ameloblasts^1^,^2^,^3^. Subsequently, enamel proteins are progressively degraded by kallikrein 4 (KLK4), which is expressed by transition- and maturation-stage ameloblasts^4^.

In addition to the enamel proteins and proteinases, transforming growth factor-β (TGF-β) isoforms are expressed by ameloblasts throughout amelogenesis^5^. TGF-β1 regulates the expression of both MMP20^6^ and KLK4 mRNAs^7^. TGF-β1 also regulates enamel mineralization and maturation through KLK4 expression^8^, and its expression is up-regulated in maturation-stage enamel organs, which might induce ameloblast apoptosis^9^. Other studies have shown that SMAD3 is required for enamel biomineralization, and TGF-β is critical for its signalling^10^. TGF-β1 over-expression in the pre-secretory stage of ameloblasts resulted in an abnormal enamel mineralization pattern^11^. Studies relevant to TGF-β for amelogenesis have mostly been conducted at the genetic level, although a few studies have been conducted at the protein level. We previously found that TGF-β1 is contained in the extracellular matrix of secretory-stage enamel and induces the differentiation of human periodontal ligament cells^12^.

In this study, we report the complicated autocrine system of TGF-β1 during enamel formation by showing the gene expression, activation, inactivation, protein-protein interactions and signalling induction of TGF-β1 during amelogenesis at both the protein and genetic levels.

## Results

### Gene expression of TGF-β1 during enamel formation

**Figure 1:**
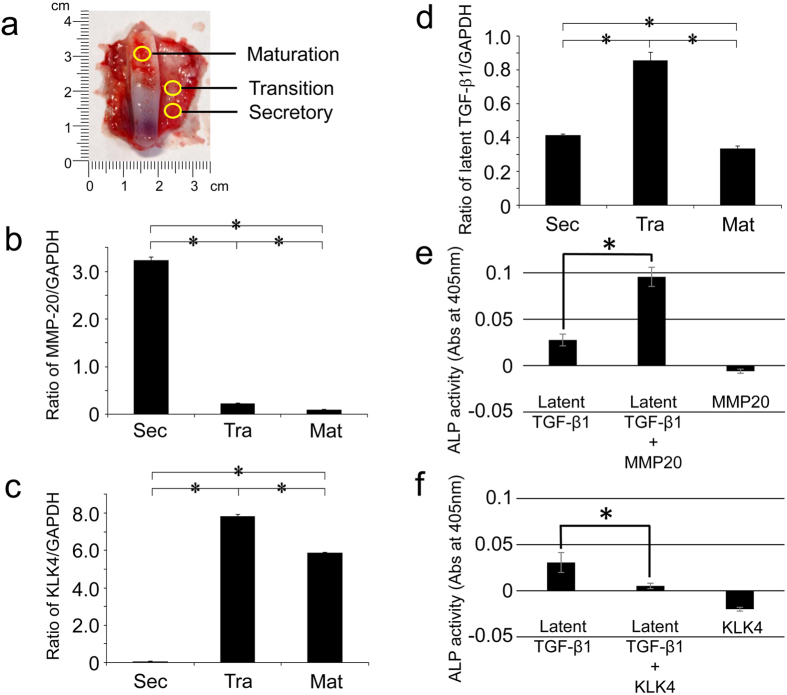
Expression of porcine MMP20, KLK4 and latent TGF-β1 in porcine ameloblasts and *in vitro* activation of the latent TGF-β1 by porcine MMP20 or KLK4. (**a**) Permanent incisor and enamel organ epithelia from a 5-month-old pig. The secretory-, transition- and maturation-stage ameloblast layers were excised with a razor blade. On the permanent incisor of 5-month-old pig, the secretory ameloblasts are separated from the enamel layer along with the rest of the enamel organ epithelia. In contrast, the maturation-stage ameloblasts are adherent to the underlying enamel layer because the basement membrane of maturation-stage ameloblasts mediates the attachment of those epithelial cells to the mineralized tooth surface. The mRNA expression was assayed by qPCR analysis of (**b**) MMP20, (**c**) KLK4 and (**d**) latent TGF-β1 in secretory (Sec)-, transition (Tra)- and maturation (Mat)-stage ameloblasts. Each ratio was normalized to a reference gene (GAPDH), and the relative quantification data of MMP20, KLK4 and latent TGF-β1 in ameloblasts were generated on the basis of a mathematical model for relative quantification in a qPCR system (n = 6 ameloblasts for each stage). The latent TGF-β1 was incubated with native pMMP20 or pKLK4. ALP-inducing activity of HPDL cells exposed to (**e**) latent TGF-β1 only, latent TGF-β1 with MMP20 and MMP20 only samples, or (**f**) latent TGF-β1 only, latent TGF-β1 with KLK4 and KLK4 only samples (n = 9 culture wells for each group).

We prepared total RNA isolated from enamel organ epithelium (EOE) corresponding to the secretory, transition and maturation stages (Fig. 1a). The relative quantification data for MMP20, KLK4 and latent TGF-β1 were normalized to glyceraldehyde 3-phosphate dehydrogenase (GAPDH). The qPCR analysis revealed that the mRNA levels for MMP20 were approximately 15-fold higher in secretory EOE than they were for transition or maturation EOE (Fig. 1b). In contrast, KLK4 transcripts were significantly higher in transition (~80 fold) and maturation (~60 fold) EOE than they were for secretory EOE (Fig. 1c). Latent TGF-β1 transcripts were detected in all three EOE stages. These levels were ~2-fold higher in transition EOE than in secretory or maturation EOE (Fig. 1d).

### *In vitro* activation of TGF-β1 by MMP20 and KLK4

We incubated recombinant human-latent TGF-β1 (rh-latent TGF-β1) with purified porcine MMP20 (pMMP20) or kallikrein 4 (pKLK4) (see Supplementary Fig. S1) and determined that the alkaline phosphatase (ALP)-inducing activity in human periodontal ligament cells is enhanced by activated TGF-β1 (ALP-human periodontal ligament fibroblast [HPDL] system). The incubation of rh-latent TGF-β1 without pMMP20 revealed only trace levels of ALP-inducing activity, while treatment with pMMP20 enhanced ALP-inducing activity (~4 fold) (Fig. 1e). In contrast, the incubation of rh-latent TGF-β1 treated with pKLK4 lost ALP-inducing activity (~1/3 fold) (Fig. 1f).

### Extraction of TGF-β1 in the enamel matrix

**Figure 2:**
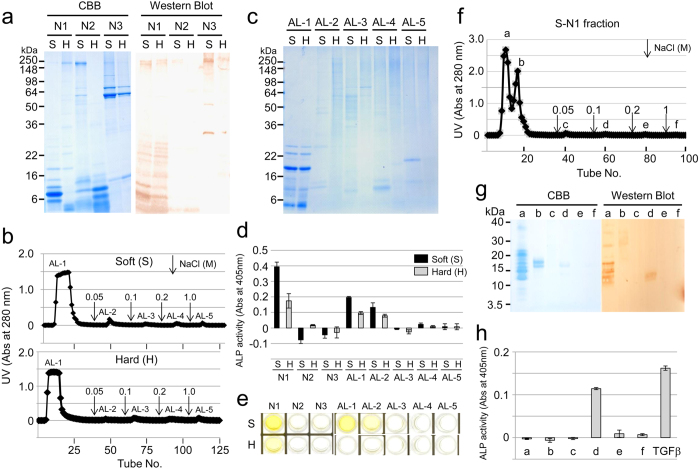
TGF-β1 in porcine enamel matrix. (**a**) SDS-PAGE (15% gel) stained with Simply Blue (CBB) (left) and western blot (right) using a specific antibody against amelogenin, showing the neutral soluble extracts from soft (S) and hard (H) enamel fractionated by successive ammonium sulfate precipitations. N1: ammonium sulfate precipitate (ASP) at 40% saturation, N2: 40–65% ASP soluble in acid and N3: 40–65% ASP insoluble in acid. (**b,f**) Heparin Sepharose chromatograms showing the absorbance at 280 nm for (**b**) AL extracts from soft enamel (~200 mg) (Soft) and hard enamel (~150 mg) (Hard), and (**f**) S-N1 extracts (~150 mg) from soft enamel. Downward-pointing arrows represent the starting points of the step gradient with 0.05, 0.1, 0.2 and 1 M NaCl. (**c**) SDS-PAGE (15% gel) stained with Simply Blue (CBB) showing fractions AL-1 to AL-5 on heparin chromatograms from soft (S) and hard (H) enamel. (**d,h**) ALP-inducing activity of HPDL cells exposed to (**d**) fractions N1 to N3 and AL-1 to AL-5 from soft (S) and hard (H) enamel, and (**h**) fractions a-f isolated from heparin chromatography. The recombinant human TGF-β1 with a carrier (0.3 ng mL^−1^) (TGFβ) was used as a positive control for the detection of ALP-inducing activity of HPDL cells (n = 9 culture wells for each sample). (**e**) ELISA for the detection of TGF-β1 capture and TGF-β1 detection antibodies in fractions N1 to N3 and AL-1 to AL-5 from soft (S) and hard (H) enamel. (**g**) SDS-PAGE (15% gel) stained with Simply Blue (CBB) (left) and western blot (right) using a specific antibody against amelogenin, showing fractions a-f isolated from heparin chromatography.

Porcine enamel matrix in soft or hard enamel was sequentially extracted under neutral (N) and alkaline (AL) conditions. The N-extracts were further fractionated into three fractions: ammonium sulfate precipitate (ASP) at 40% saturation (N1), 40–65% ASP soluble in acid (N2) and 40–65% ASP insoluble in acid (N3). Western blot analysis with an amelogenin antibody revealed that neutral soluble amelogenins were mostly fractionated in N1 (Fig. 2a). Because TGF-β1 was found in AL extracts in our previous study^12^ and because it possesses a heparin-binding site (see Supplementary Fig. S2), the AL-extracts were subjected to affinity chromatography using a heparin column. Heparin chromatography fractionated the extracts into five peaks (Fig. 2b), and SDS-PAGE showed that major amelogenins, such as P173 (25 kDa), P162 (23 kDa), and P148 (20 kDa) amelogenins, and tyrosine-rich amelogenin peptide (TRAP or 5.3 kDa amelogenin) in the AL-extracts from the soft and hard enamel eluted in the first (AL-1) peak (Fig. 2c). The N1, AL-1 and AL-2 fractions containing amelogenins enhanced ALP-inducing activity in HPDL cells, and the activity in the soft enamel was higher than that in the hard enamel (Fig. 2d). Sandwich enzyme-linked immunosorbent assay (ELISA) against both capture and detection antibodies of TGF-β1 showed that the S-N1, H-N1, S-AL-1 and S-AL-2 fractions were positive against two TGF-β1 antibodies (Fig. 2e).

Because the ALP-inducing activity in HPDL cells was highly detected in S-N1 fractions, we further fractionated it into six peaks (Fig. 2f). Although most of the amelogenin cleavage products containing P103 amelogenin were eluted in the first fraction (Fig. 2g), a small quantity of P103 amelogenin was also eluted in the fourth fraction, meaning it had some binding affinity for heparin. This fraction enhanced ALP-inducing activity in HPDL cells (Fig. 2h).

### Identification of the amelogenin-TGF-β1 complex *in vivo*

**Figure 3:**
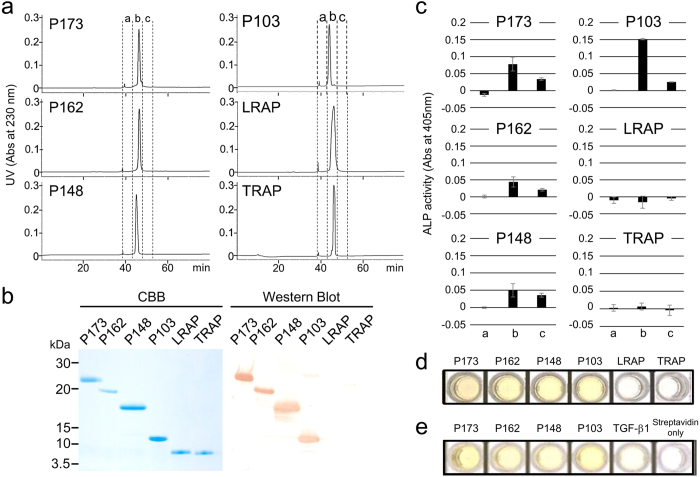
Isolation and identification of TGF-β1-unbound and TGF-β1-bound amelogenin cleavage products. (**a**) RP-HPLC chromatograms showing the absorbance at 230 nm for P173, P162, P148, P103, LRAP and TRAP amelogenins. (**b**) SDS-PAGE (15% gel) stained with Simply Blue (CBB) (left) and western blots (right) using a specific antibody against amelogenin, showing each purified amelogenin isolated by RP-HPLC. (**c**) ALP-inducing activity of HPDL cells exposed by fractions a, b and c in each amelogenins eluted by RP-HPLC (n = 9 culture wells for each sample). (**d**) ELISA for the detection of TGF-β1 capture and TGF-β1 detection antibodies. (**e**) ELISA for the detection of TGF-β1 capture and biotinylated-amelogenin detection antibodies.

To characterize the interaction between amelogenins and TGF-β1 *in vivo*, we purified P173, P162, P148, P103, TRAP (see Supplementary Figs S3 and S4), and commercially synthesized leucine-rich amelogenin peptide (LRAP) by reversed phase high-performance liquid chromatography (RP-HPLC) (Fig. 3a). Each purified amelogenin was observed as a single band on SDS-PAGE and was confirmed as an amelogenin by western blot analysis using the amelogenin antibodies against the P173, P162, P148 and P103 amelogenins (Fig. 3b). Because neither tyrosine-rich amelogenin peptide (TRAP) nor LRAP were detected by the amelogenin antibody, we characterized them by liquid chromatography–tandem mass spectrometry (LC-MSMS) analysis (see Supplementary Fig. S5). ALP-inducing activity in HPDL cells was detected for the P173, P162, P148 and P103 amelogenins, with the P103 amelogenin possessing the highest activity compared to the other three amelogenins (Fig. 3c). No ALP-inducing activity was detected in LRAP and TRAP. The amount (ng) of TGF-β1 bound to the amelogenin was calculated from the standard TGF-β1 by ELISA (see Supplementary Fig. S6). The P173, P162, P148 and P103 amelogenins were positive against both capture and detection antibodies of TGF-β1 (Fig. 3d) and contained approximately 1–4 ng of TGF-β1 per mg of amelogenin. To exclude the possibility of co-purification (i.e., co-elution) of TGF-β1 and amelogenin by RP-HPLC, we performed a modified sandwich ELISA. We combined each amelogenin with the capture antibody of TGF-β1 coated on a plate, and we labelled the biotinylated amelogenin antibody as the detection antibody (see Supplementary Fig. S7). The positive signal against horseradish peroxidase (HRP)-conjugated streptavidin was observed in P173, P162, P148 and P103 amelogenins but not in control (Fig. 3e). We interpret these findings as evidence of TGF-β1 binding to four amelogenins, P173, P162, P148, and P103, *in vivo*.

### *In vitro* binding experiments of amelogenin and TGF-β1

We attempted to gain more information about the binding of amelogenins using *in vitro* experiments (see Supplementary Fig. S8). Total amounts (ng) of original or bound TGF-β1 in fractions obtained from RP-HPLC before and after *in vitro* binding experiments against one mg of P173, P162, P148 and P103 amelogenins were calculated from the standard carrier free-human TGF-β1 (CF-hTGF-β1) (0.3 ng mL^−1^), as shown in Table 1. The original P173, P162, P148 and P103 amelogenins retained *in vivo* TGF-β1 activity during RP-HPLC, and the amounts of TGF-β1 after the dilution correction were 0.345 ± 0.037 ng mg^−1^ of P173, 0.201 ± 0.024 ng mg^−1^ of P162, 0.263 ± 0.096 ng mg^−1^ of P148 and 0.797 ± 0.076 ng mg^−1^ of P103. During the *in vitro* binding experiments, four amelogenins were able to bind CF-hTGF-β1, and the amount of TGF-β1 bound to the amelogenins after dilution correction was at nearly equal levels (1.062–1.176 ng mg^−1^ of amelogenins).

We also tested if the rh-latent TGF-β1 is able to bind to P173 and/or P103 amelogenins using the *in vitro* binding experiment. There was no significant difference in ALP-inducing activities before and after the binding (see Supplementary Fig. S9).

### Dynamic light scattering (DLS)

**Figure 4:**
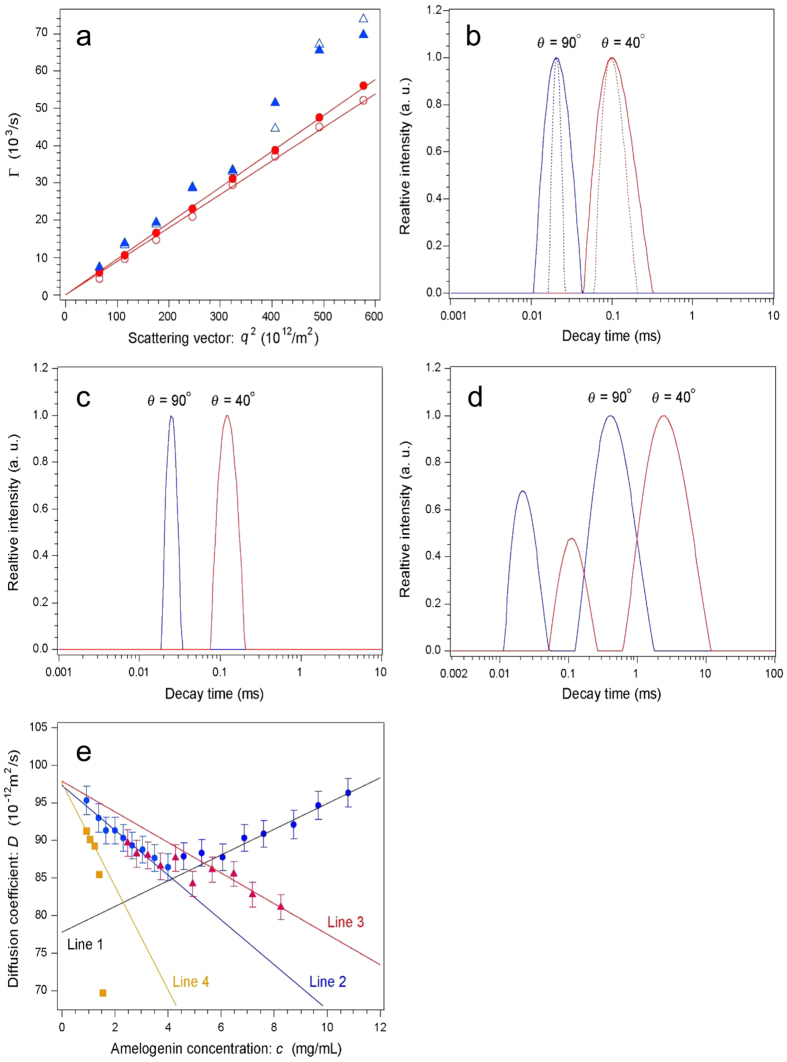
Dynamic light scattering analysis of P103 amelogenin-TGF-β1 complex. (**a**) Γ vs. *q*^2^ plot at high (10.8 mg mL^−1^, closed symbols) and low (2.31 mg mL^−1^, open symbols) *c* for the original P103 amelogenin. Circles correspond to data analysed using the intensity-percentage CONTIN method, and triangles correspond to data analysed using the mass-percentage method. Good correlation was observed for intensity-percentage results. (**b**) Decay time distributions at θ = 40° (red) and 90° (blue) analysed using the CONTIN (solid curve) and NNLS (dotted curve) methods at *c* = 9.66 mg mL^−1^ for original P103 amelogenin. (**c**) Decay time distributions at θ = 40° (red) and 90° (blue) analysed using the CONTIN method at *c* = 8.25 mg mL^−1^ for P103-1 amelogenin. (**d**) Decay time distributions at θ = 40° (red) and 90° (blue) analysed using the CONTIN method at *c* = 1.41 mg mL^−1^ for fast mode of P103-2 amelogenin. (**e**) *D* vs. *c* relationship for molecules of original P-103 amelogenin (Line 1 and 2), P103-1 amelogenin (Line 3) and P103-2 amelogenins (Line 4) in solution.

Because the binding of TGF-β1 predominantly occurred with P103 amelogenin *in vivo*, we focused on this amelogenin and investigated the molecular weight of P103 amelogenin-TGF-β1 complexes by DLS analysis. Using RP-HPLC, we purified two TGF-β1-unbound P103 amelogenins (P103-1 and -2 amelogenins) in the first eluate isolated by heparin affinity chromatography from the S-N1 sample (Fig. 2f and see Supplementary Fig. S10). We compared this sample to TGF-β1-bound P103 amelogenin (original P103 amelogenin) using DLS analysis. Figure 4a shows the Γ vs. *q*^2^ plot at high (10.8 mg mL^−1^, closed symbols) and low (2.31 mg mL^−1^, open symbols) *c* for the original P103 amelogenin. The circles correspond to the data analysed using the intensity-percentage CONTIN method, and the triangles correspond to the data analysed using the mass-percentage method. Good correlations with lines passing through the origin were observed for the intensity-percentage plot, while the correlations were poor for the mass-percentage plot and were particularly poor in the high *q*^2^ region, suggesting that scattering from very small particles affected the results. The deviation was enhanced at very low *c* (<1.5 mg mL^−1^), meaning it could be attributed to scattering from Tris molecules. Therefore, the intensity-percentage analysis for the Γ calculation was used. The same analysis was performed for the P103-1 and -2 amelogenins. Figure 4b shows the decay time (1/Γ) distribution at *c* = 9.66 mg mL^−1^ analysed using the CONTIN (solid curve) and NNLS (dotted curve) methods at θ = 40° and 90° for the original P103 amelogenin. The analyses produced virtually identical results (Δ < 2%). Because the decay time distribution was proportional to the *R*_H_ distribution of amelogenin molecules, the broad distribution profiles indicate particle polydispersion (polydispersion index = 0.223 at θ = 40°). This feature was independent of *c*. Aggregation was frequently observed at low *c* (<1.5 mg mL^−1^), which hindered the precise measurement of the average molecular weight of particles using static light scattering. It was difficult to distinguish whether the aggregates originated from the protein molecules or from dust particles, which were relatively abundant, due to the low scattering intensity of the protein molecules. Figure 4c shows the decay time distribution at *c* = 8.25 mg mL^−1^ analysed using the CONTIN method at θ = 40° and 90° for heparin-treated sample 1. The distribution profile was much narrower (polydispersion index = 0.08 at θ = 40°) than that of the heparin-untreated sample. Figure 4d shows the decay time distribution at *c* = 1.41 mg mL^−1^ for the P103-2 sample. There were two peaks: fast and slow decay times, which corresponded to small and large particles, respectively. The average size of the smaller particles was approximately 1/300 of the larger particles. Figure 4e shows the *D* vs. *c* relationship for amelogenin molecules calculated using Eq. (2) in “Dynamic light scattering” in the Supplementary Note. The blue circles, red triangles, and ochre squares correspond to the data points for the original P103 amelogenin, P103-1 amelogenin, and P103-2 amelogenin (fast mode), respectively. The error for each data point was less than 2%. Note that *D* did not change monotonically with *c* for the heparin-untreated sample. At *c* > 6.0 mg mL^−1^, the data points lay on a correlation line with a positive slope (Line 1). From 4.0 to 6.0 mg mL^−1^, *D* did not depend on *c*, and the data points fall on a line with a negative slope (Line 2) at *c* < 4.0 mg mL^−1^. The extrapolation of Line 1 intersects the vertical axis at *D*_0_ = (77.8 ± 1.2) × 10^−12^ m^2^ s^−1^, corresponding to an *R*_H_ of 3.21 ± 0.04 nm after a viscosity correction (0.89 cP). The extrapolation of Line 2 intersects the vertical axis at *D*_0_ = (97.3 ± 0.7) × 10^−12^ m^2^ s^−1^, corresponding to an *R*_H_ of 2.57 ± 0.02 nm. The *D* of P103-1 amelogenin also exhibited two dependences on *c*. The data fell on a line with a negative slope (Line 3) at *c* > 6.0 mg mL^−1^, which became a line with a steeper negative slope at *c* < 4 mg mL^−1^ that coincided with Line 2 for the original P103 amelogenin. Note that the extrapolation of Line 3 intersects the vertical axis at the same point as that of Line 2.

### Extraction of TGFBR1 in enamel organ epithelia and the interaction of amelogenin-TGF-β1 complex and TGFBR1

**Figure 5:**
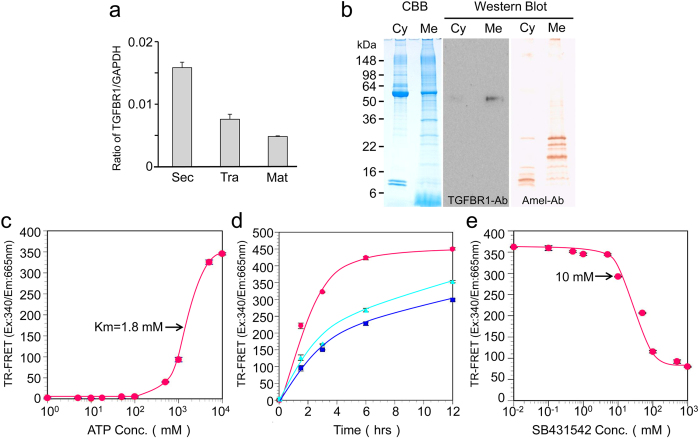
TGFBR1 expression and extraction in porcine incisor enamel organ epithelia and TR-FRET TGFBR1 kinase assay. (**a**) TGFBR1 mRNA expression by qPCR analysis in secretory (Sec)-, transition (Tra)- and maturation (Mat)-stage ameloblasts. Each ratio was normalized to a reference gene (GAPDH) to generate relative quantification data of TGFBR1 in ameloblasts and was analysed on the basis of a mathematical model for relative quantification in the qPCR system (n = 6 ameloblasts for each stage). (**b**) SDS-PAGE (15% gel) stained with Simply Blue (CBB) showing both cytosol (Cyt) and membrane (Mem) fractions. Western blots showing each fraction using specific antibodies against TGFBR1 (TGFBR1-Ab) and amelogenin (Amel-Ab). TGFBR1 in the enamel organ epithelia was incubated with 100 nM of ULight-Topo IIa (Thr1342) peptide as the substrate in kinase buffer. (**c**) ATP titration curve: Serial dilutions of ATP ranging from 1 μM to 10 mM were added to the substrate (n = 5 wells for each concentration of sample). (**d**) Enzymatic time course: TGFBR1 was incubated with substrate and 5 mM ATP (blue) upon the addition of 0.5 μg of CF-hTGF-β1 (red) or 0.1 mg of P103 amelogenin-TGF-β1 complex (green) for 1.5, 3, 6 and 12 h at 37 °C (n = 5 wells for each concentration of sample). (**e**) Enzyme inhibition curve: Serial dilutions of SB431542 ranging from 10 nM to 1 mM were incubated with the substrate and 5 mM ATP. In all experiments, kinase reactions were terminated by the addition of 10 mM EDTA (n = 5 wells for each concentration of sample).

To learn more about the signalling induction function of the amelogenin-TGF-β1 complex during enamel formation, we performed a qPCR assay to gain quantitative information on TGFBR1 gene expression. We normalized the relative quantification data for TGFBR1 in three EOE to a reference gene (GAPDH) (Fig. 5a). The qPCR analysis showed the mRNA levels of TGFBR1 transcript that were observed in the three EOE stages. In secretory EOE, these levels were higher (~2 fold) than those in the transition and maturation EOE.

We isolated TGFBR1 from enamel organ epithelia to test the signalling induction of TGF-β1 *in vitro*. An immunopositive band was identified with a TGFBR1 antibody at approximately 53 kDa in the membrane protein fraction (Fig. 5b). Interestingly, immunopositive bands were detected with an amelogenin antibody in both the cytosolic and membrane fractions.

The kinase assay for TGFBR1 was performed using time-resolved fluorescence resonance energy transfer (TR-FRET). The TR-FRET signal occurred between an ULight-Topo IIα peptide that could be phosphorylated by TGFBR1 and a europium-anti-phospho-antibody that can only bind to phosphate residues on a peptide. By analysing this signal, the effect of ATP concentration on TGFBR1 activity was investigated. The TR-FRET assay showed an equivalent apparent *K*_m_ concentration for ATP at 1.8 mM (Fig. 5c). A concentration of 5 mM ATP (~2.8x apparent *K*_m_) was selected, and the enzyme progress curve was linear for up to 12 h with or without the addition of 30 μg of P103 amelogenin-TGF-β1 complex or 250 ng mL^−1^ of CF-hTGF-β1 (Fig. 5d). After 12 h, the FRET signal with the addition of CF-hTGF-β1 was approximately 1.4 fold higher than that of the control (i.e., without the addition of P103 amelogenin-TGF-β1 complex or CF-hTGF-β1). The addition of the P103 amelogenin-TGF-β1 complex enhanced the FRET signal, although it was only ~5% higher than the control. We also performed enzyme inhibition experiments with SB431542, a specific and selective inhibitor for TGFBR1. The SB431542 inhibited TGFBR1 activity in a concentration-dependent manner at 10 μM in the TR-FRET assay (Fig. 5e).

### *In vitro* inactivation of TGF-β1 by MMP20 and KLK4

**Figure 6:**
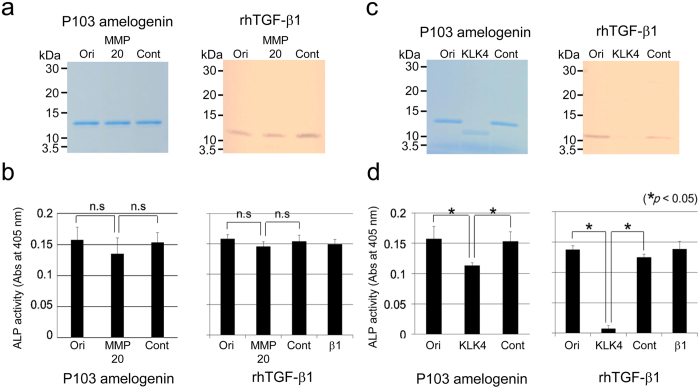
Digestion of P103 amelogenin-TGF-β1 complex and CF-hTGF-β1 by porcine MMP20 or KLK4. Purified P103 amelogenin-TGF-β1 complex and CF-hTGF-β1 were incubated with porcine MMP20 or KLK4 for 20 h, respectively. (**a,c**) SDS-PAGE (15% gel) stained with Simply Blue (CBB) showing the P103 amelogenin-TGF-β1 complex (P103 amelogenin) and with silver showing CF-hTGF-β1 (rhTGF-β1). (**b,d**) The ALP-inducing activity of HPDL cells exposed to the P103 amelogenin-TGF-β1 complex (P103 amelogenin) and CF-hTGF-β1 (rhTGF-β1) samples treated with pMMP20 or pKLK4 (n = 9 culture wells for each sample). Ori: original sample (i.e., no incubation), MMP20: incubation with pMMP20, KLK4: incubation with pKLK4, Cont: incubation without pMMP20 or pKLK4 and β1: recombinant human TGF-β1 with a carrier (0.3 ng mL^−1^).

Because rh-latent TGF-β1 lost ALP-inducing activity after incubation with KLK4 (Fig. 1f), we digested the P103 amelogenin-TGF-β1 complex and CF-hTGF-β1 with MMP20 or KLK4. Neither the P103 amelogenin-TGF-β1 complex nor CF-hTGF-β1 was degraded by MMP20, which would have been detected at approximately 13 or 12 kDa following digestion (Fig. 6a). There was no significant difference in ALP-inducing activity before and after digestion (Fig. 6b). In contrast, both the P103 amelogenin-TGF-β1 complex and CF-hTGF-β1 were degraded by KLK4, which reduced the apparent sizes of those proteins to approximately 12 kDa, with no detectable size on SDS-PAGE (Fig. 6c). The level of ALP-inducing activity dropped to approximately 70% in the P103 amelogenin-TGF-β1 complex and 8% in CF-hTGF-β1 compared to the levels before digestion (Fig. 6d).

## Discussion

MMP20 is expressed by secretory-stage ameloblasts^1^,^2^,^3^, while KLK4 is specifically expressed by transition- and maturation-stage ameloblasts^2^,^13^. Our qPCR analysis revealed that the MMP20 mRNA expression level was significantly higher in secretory EOE, while the KLK4 mRNA expression level was significantly higher in transition- and maturation-EOE. These data confirm the accuracy of the dissections to obtain samples of the three stages of EOE from permanent incisors.

In rodents, TGF-β is expressed in developing and mature ameloblasts and is also detected in inner dental epithelium before enamel matrix secretion^14^,^15^. Moreover, TGF-β1 is expressed during differentiation of the enamel organ and initiation of matrix secretion in human teeth^5^. We demonstrated that the latent TGF-β1 transcript was expressed throughout enamel formation, although its mRNA expression level in transition EOE was significantly higher than in both secretory- and maturation-stage EOE. This finding led us to suspect that latent TGF-β1 is activated in the enamel matrix.

In general, TGF-β is synthesized as a precursor, as it contains a propeptide domain with a TGF-β homodimer^16^ that forms the latent TGF-β complex by interacting with a latency-associated peptide (LAP) after synthesis^17^. TGF-β activation is caused by several factors, such as pH^18^, reactive oxygen species^19^, thrombospondin-1^20^ and integrin^21^,^22^,^23^,^24^. In addition to these factors, proteases such as plasmin, MMP2 and MMP9 are also able to activate TGF-β through proteolytic degradation of the latent TGF-β complex^25^,^26^. We demonstrated that the level of ALP-inducing activity dramatically increased after incubation of rh-latent TGF-β1 with MMP20, although KLK4 was able to reduce the TGF-β1 activity.

During enamel formation, both MMP20 and KLK4 are necessary to process and degrade proteins from the enamel matrix of developing teeth. We previously showed that MMP20 is able to activate proKLK4^27^. In addition to enamel proteins, junctional complexes present on ameloblasts may also be cleaved by MMP20 to foster cell movement, which is necessary for the formation of the decussating enamel rod pattern^28^,^29^. Considering that TGF-β signalling is partially mediated by regulating MMP20 mRNA expression^6^, the present data suggest that autocrine regulation of TGF-β1 through MMP20 coincides with other reactions related to MMP20 in the secretory-stage enamel.

In mammals, three TGF-β isoforms (TGF-β1 to -β3) have been identified, each having similar functions *in vitro*^30^,^31^. Three TGF-β isoforms are expressed in human ameloblasts at high levels, where TGF-β1 > TGF-β2 > TGF-β3^5^. TGF-β1 has been demonstrated to possess strong heparin-binding activity *in vitro*^32^. Heparin and highly sulfated heparin sulfate enhanced TGF-β1 activity; however, they had no effect on TGF-β2 and TGF-β3 activities^33^. We found that the mature TGF-β1 possesses one heparin-binding site (Arg^372^-Lys^375^) that is not present in mature TGF-β2 or TGF-β3 (see “Extraction of TGF-β in enamel matrix” in the Supplementary Note). Based on this information, we attempted to isolate N1 extracts with heparin affinity chromatography and were able to detect TGF-β with a small quantity of P103 amelogenin in a fraction that had binding affinity for heparin. In contrast, TGF-β in AL extracts was detected with other major amelogenins, including P173, P162, P148 and TRAP, in a fraction having no binding affinity for heparin. This discovery raised the possibility of different TGF-β isoforms in the N1 and AL extracts. However, we demonstrated that both N1 and AL extracts were positive against the TGF-β1 antibody by ELISA. This finding may suggest that TGF-β1 interacted with amelogenin cleavage products, which may have influenced their extraction. In addition, we also demonstrated that TGF-β1 activity in the soft enamel was higher than that in the hard enamel. This finding suggests that TGF-β1 may reduce its activity upon the degradation of the enamel matrix components during the maturation stage.

Amelogenin is the major secretory product of ameloblasts and is critical for proper tooth enamel formation. We have previously demonstrated that MMP20 catalyses the cleavage reactions that generates the P173 and LRAP cleavage products. These products accumulate in secretory-stage enamel^34^. These cleavage reactions generate the most abundant amelogenin cleavage products in the secretory-stage enamel: P148, TRAP, P162 and P103 amelogenins. Therefore, we focused on six amelogenins in our investigations of protein-protein interactions with TGF-β1: P173, P162, P148, P103, LRAP and TRAP. The present study demonstrated that the purified P173, P162, P148 and P103 amelogenins enhanced ALP-inducing activity in HPDL cells. This finding led us to consider that TGF-β1 may either bind to or co-elute on RP-HPLC with those amelogenins.

In this study, we modified our method to qualitatively identify the amelogenin-TGF-β1 complex using a TGF-β1 antibody as the capture antibody and a biotinylated amelogenin antibody as the detection antibody (see “Identification of amelogenin-TGF-β1 complex *in vivo*” in the Supplementary Note). We demonstrated that the P173, P162, P148 and P103 amelogenin samples were able to form antigen-antibody complexes not only between two layers of the TGF-β1 antibody but also between the TGF-β1 antibody and the biotinylated amelogenin antibody. These findings suggest that TGF-β1 in the secretory-stage enamel binds to the P173, P162, P148 and P103 amelogenins. The full-length amelogenin is a complex molecule consisting of four functional domains: TRAP, Coil, a PXX repeat and a hydrophilic C-terminal domain^35^,^36^ (see Supplementary Fig. S11). Considering that neither TRAP nor LRAP enhanced ALP-inducing activity in HPDL cells, the present study suggests that the TGF-β1 binding site may be present on the coil- or PXX-domains of amelogenin.

Amelogenin assembles into spherical structures called “nanospheres”^37^,^38^,^39^ or “micelles”^40^ through a hierarchical process^41^,^42^. At physiological pH, P173, P162 and P148 amelogenins assemble into highly organized oligomers and nanospheres that bind together to form branched chains^39^,^41^,^42^,^43^. Following the processing of P173, P162 and P148 amelogenins by MMP20, the generated P103 amelogenin becomes a rod-like structure in neutral solution^40^. We demonstrated that the amount of TGF-β1 bound to the P103 amelogenin *in vivo* was approximately 2.3- to 4.0-fold higher than that bound to the other three amelogenins, and those four amelogenins possessed almost equal binding capacity for TGF-β1 *in vitro*. These findings suggest that TGF-β1 can easily bind to the rod-like structure of P103 amelogenin after the C-terminal and TRAP domains were cleaved off by MMP20.

DLS analysis has been used to investigate the mechanisms of amelogenin-cell interactions during amelogenesis^36^,^44^. Porcine secretory-stage enamel contains 50% water, 25% minerals and 25% proteins, although those ratios are different in the outer, outer-inner and inner layers^40^. The P173, P162 and P148 amelogenins bind to each other via hydrophobic interactions to form the larger aggregates, which form a precipitate in neutral solution, while P103 amelogenin is soluble^40^. Therefore, we focused on determining the size distribution profile of the P103 amelogenin-TGF-β1 complex by DLS to understand its state in solution.

In the positive *D* vs. *c* relationship (Fig. 4e), the solution of the original P103 amelogenin (Line 1) at *c* > 6.0 mg mL^−1^ indicates that a repulsive intermolecular interaction was dominant, whereas the negative relationship (Line 2) at *c* < 4.0 mg mL^−1^ indicates a predominantly attractive force between the molecules. If the observed particles are all the same species independent of *c*, this feature would not be expected, i.e., the particles must adopt multiple states, meaning that there were likely variable extents of oligomerization in the solution. Because the scattering intensity is approximately proportional to six times *R*_H_ and proportional to the number of particles, smaller particles in the solution become evident at low *c*, which are conditions when they are the major species. The results for the original P103 amelogenin indicate that the particles corresponding to an *R*_H_ of 2.57 ± 0.02 nm were the dominant state in solution.

Compared the original P103 amelogenin and the two TGF-β1-unbound P103 amelogenins (P103-1 and -2 amelogenins), the inclination of the correlated line in the *D* vs. *c* relationship for the solution of P103-1 amelogenin (Line 3) is close to that of Line 2. In addition, these lines’ extrapolations converge at the same *D*_0_, suggesting that isomers with slightly different surface charges coexisted in this solution.

As described in the previous section, the original P103 amelogenin showed activity against TGF-β1, whereas P103-1 amelogenin did not. Because the *D*_0_ obtained from the original P103 amelogenin using the data in the low *c* region (intersect of Line 2) coincided with that for the P103-1 and -2 amelogenins (intersects of Lines 3 and 4), the *R*_H_ of 2.57 ± 0.02 nm likely corresponds to the particle radius of pure amelogenin molecules. In contrast, the *D*_0_ obtained for the original P103 amelogenin using the data in the high *c* region (intersect of Line 1) means that the *R*_H_ of 3.21 ± 0.04 nm likely corresponds to the complex of amelogenin and TGF-β1, as only Line 1 indicates repulsive intermolecular interaction.

Because the measured *R*_H_ of 2.57 ± 0.02 nm was much larger than that expected from the molecular weight (*M*_w_) of an amelogenin monomer, 12.7 kDa, the particle *M*_w_ was estimated using the experimentally established relationship between the *R*_H_ and *M*_w_ of protein molecules. When the protein molecules have a native folding state, the following relationship is observed^45^.


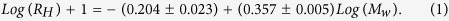


That is, a particle with *R*_H_ = 2.57 nm would have an average *M*_w_ of 33.2 kDa, which is very close to the 37.1 kDa of an amelogenin trimer^46^. A particle with *R*_H_ of 3.21 ± 0.04 nm had an average *M*_w_ of 61.8 kDa, which corresponds to the binding state of an amelogenin trimer and an active TGF-β homodimer (25.0 kDa) with a molar ratio of 1:1.

Immunohistochemical analysis in developing murine teeth has shown that TGFBR1 expression occurs at the apical ends of secretory-stage ameloblasts interfaced with the enamel matrix^15^. The present study demonstrated that TGFBR1 and TGF-β1 transcripts were expressed throughout enamel formation by our qPCR analysis. These findings are in agreement with previous reports that both TGF-β1 and TGFBR1 are expressed during differentiation of the enamel organ and initiation of matrix secretion in human and murine teeth^5^,^15^.

TGFBR1 is a transmembrane receptor that possesses a serine/threonine kinase domain, which mediates signalling the family of TGF-β ligands^47^. Using EOE at the secretory stage, we were able to extract and identify TGFBR1 having an approximate molecular weight of 53 kDa in the membrane and membrane-associated protein fractions. When we used this TGFBR1 fraction as a control for an *in vitro* kinase assay by TR-FRET, the FRET signal was increased during the incubation, suggesting the induction of TGF-β signalling via the endogenous TGFBR1 in EOE. Our *in vitro* kinase assay further demonstrated that the addition of the P103 amelogenin-TGF-β1 complex weakly enhanced the FRET signal. This finding suggests that P103 amelogenin may be necessary for transporting TGF-β1 to its receptor on ameloblasts to induce TGF-β signalling.

Enamel proteins are progressively degraded by KLK4 within the enamel matrix so the enamel crystallites can grow in width and thickness. The cleavage products are then reabsorbed into ameloblasts during the maturation stage^34^,^40^,^48^,^49^. We previously showed that KLK4, which was activated by MMP20, cleaves the catalytic domain of MMP20 at physiologic pH under smooth-ended ameloblasts^27^. The present study revealed that MMP20 was not able to cleave the P103 amelogenin-TGF-β1 complex or CF-hTGF-β1, while KLK4 was able to degrade them. When only CF-hTGF-β1 was incubated with KLK4, approximately 8% of its activity remained. P103 amelogenin was able to rescue the loss of TGF-β1 activity, and approximately 70% of its activity was retained by binding to P103 amelogenin. Thus, our findings indicate that P103 amelogenin is necessary for maintaining TGF-β1 activity. Considering that TGF-β1 induces the expression of KLK4 mRNA^7^, our present findings suggest that TGF-β1 is able to induce KLK4 production and that activated KLK4 is able to inactivate the TGF-β1 activity in the maturation stage of enamel.

**Figure 7:**
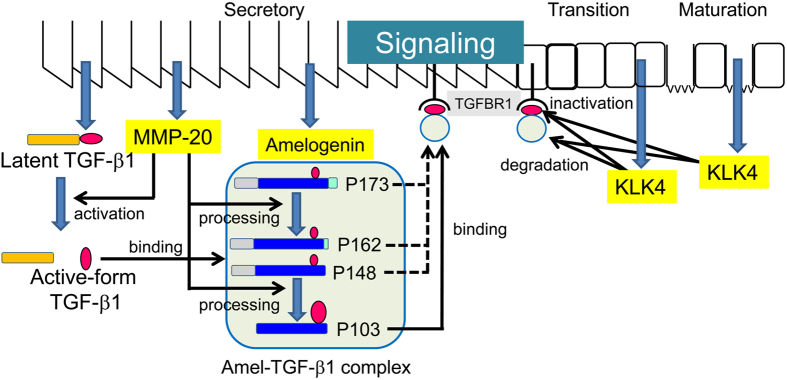
Autocrine system of TGF-β1 signalling in the enamel matrix.

We summarize the autocrine system of TGF-β1 signalling in the enamel matrix in Fig. 7. TGF-β1 is synthesized and secreted into the secretory-stage enamel matrix as the latent TGF-β1 complex. Latent TGF-β1 is activated by MMP20, coinciding with the processing of amelogenins by the same proteinase. The activated TGF-β1 binds to amelogenin cleavage products, mostly the neutral-soluble P103 amelogenin, to maintain its activity. The P103 amelogenin-TGF-β1 complex binds to TGFBR1 to induce TGF-β1 signalling. The P103 amelogenin-TGF-β1 complex is slowly cleaved by KLK4, which is secreted into the transition- and maturation-stage enamel matrix, which reduces its activity.

To exert the multiple biological functions of TGF-β1 during amelogenesis, we propose that TGF-β1 is activated or inactivated by MMP20 or KLK4 and that the amelogenin cleavage product is necessary for the in-solution mobility of TGF-β1, which is necessary for binding to its receptor on ameloblasts and retention of its activity.

## Methods

All experimental procedures involving the use of animals were reviewed and approved by the Ethics Committee of the Institute of Tsurumi University, Yokohama, Japan. The methods were carried out in accordance with the relevant guidelines and regulations. Tooth germs of permanent molars for protein study and permanent incisors for genetic study were surgically extracted from the mandibles of deceased 5-month-old pigs from the Meat Market of the Metropolitan Central Wholesale Market (Shinagawa, Tokyo, Japan).

### Preparation of tooth germ cells

Secretory and transition EOE were dissected from the inner surface of the removed enamel organ epithelia, and maturation EOE were prepared from the labial surface of the incisor (Fig. 1a).

### Quantitative real-time PCR

Quantitative real-time PCR (qPCR) for the total RNAs of the EOE at three stages was performed using the SYBR Green technique on a LightCycler Nano (Roche Diagnostics, Mannheim, Germany) (see “Quantitative real-time PCR” in the Supplementary Methods).

### Preparation and extraction of soft and hard enamel

The EOE and dental pulp tissues were removed from the tooth germs of permanent molars using tissue forceps. The soft, cheese-like enamel and the remaining hard, chalky-like enamel were separated from the crowns using a spatula. The soft and hard enamel shavings were separately homogenized in 50 mM Sörensen buffer (pH 7.4) containing a protease inhibitor cocktail (Sigma/Aldrich, St. Louis, MO, USA). The soluble fraction (producing the N-extracts) was collected by centrifugation. Insoluble material was further homogenized in 50 mM carbonate-bicarbonate buffer (pH 10.8) containing the protease inhibitor cocktail, (Sigma/Aldrich) and the soluble fraction (AL extracts) was collected by centrifugation.

### Ammonium sulfate fractionation of enamel proteins in N-extract

N-extracts obtained from both the soft (S) and hard (H) enamel were raised to 40% saturation by the addition of ammonium sulfate. The precipitates (S-N1 or H-N1 fractions) were removed by centrifugation. The supernatants were raised to 65% saturation, and the precipitates were pelleted by centrifugation. The 40–65% saturation pellets were resuspended in 5 mL of 0.5 M acetic acid, and the supernatants (S-N2 and H-N2) and precipitates (S-N3 and S-H3) were separated by centrifugation, respectively. All samples were characterized by the ALP-HPDL system and ELISA.

### Heparin affinity chromatography

The AL-extracts obtained from the soft and hard enamel (S-AL and H-AL) were buffer-exchanged into 50 mM Tris–HCl and 6 M urea (pH 7.4) with a YM-3 membrane (Merck KGaA, Darmstadt, Germany). The sample was applied onto a Heparin Sepharose 6 Fast Flow column (1.6 cm × 20 cm, GE Healthcare, Uppsala, Sweden) with buffer A: 50 mM Tris–HCl and 6 M urea (pH 7.4). Proteins were eluted with a step gradient of NaCl (0, 0.05, 0.1, 0.2 and 1 M) in buffer A at a flow rate of 0.2 mL min^−1^ at 4 °C while monitoring the absorbance at 280 nm. Each fraction was dialyzed against water and characterized by the ALP-HPDL system and ELISA.

### Isolation of porcine matrix metalloprotease 20 (pMMP20) and kallikrein 4 (pKLK4)

The pMMP20 fraction was obtained from the second fraction eluted in the heparin chromatography described above, while the pKLK4 was prepared from the H-N2 extracts (see “Isolation of porcine kallikrein 4” in Supplementary Methods). Purified pMMP20 or pKLK4 were characterized by zymography with casein or gelatin zymogel, respectively (see “Enzymograms” in Supplementary Methods).

### *in vitro* activation of TGF-β1 by MMP20 or KLK4

The rh-latent TGF-β1 (1 μg) (Cell Signaling Technology, Danvers, MA, USA) was dissolved with 50 μL of 50 mM Tris-HCl and 10 mM CaCl_2_ (pH 7.4) and incubated with pMMP20 or pKLK4 (approximately 1 μg each) for 20 h at 37 °C. Reaction aliquots at 0 and 20 h were characterized using an alkaline phosphatase-human periodontal ligament cell-line (ALP-HPDL) system.

### Purification of porcine amelogenins

The following nomenclature is used to describe porcine amelogenins: P173 is the uncleaved 173-amino-acid major amelogenin; and P162, P148, P103, and P45 are its N-terminal cleavage products. P45 is also known as the TRAP. P56 is an amelogenin translated from an alternatively spliced mRNA transcript and is known as the LRAP^50^. The unbound fraction (i.e., the eluate against 0 M NaCl) obtained from heparin chromatography of S-AL extracts was fractionated on a Sephadex G-100 column, and the fourth and eighth fractions were further isolated by RP-HPLC using a Discovery C18 column (4.6 mm × 5 cm, Sigma-Aldrich/Supelco, Bellefonte, PA, USA). In addition, the S-N1 fraction was used for the purification of P103 amelogenin. The P56 amelogenin (LRAP) was commercially synthesized (Yenzyme, South San Francisco, CA, USA). All amelogenins (P173, P162, P148, P103, TRAP and LRAP) were finally purified by RP-HPLC using the same Discovery C18 column and were eluted with a linear gradient (40 to 65% B in 60 min) at a flow rate of 1.0 mL min^−1^. Buffer A was 0.05% trifluoroacetic acid (TFA); buffer B was 0.1% TFA in 80% aqueous acetonitrile. Protein was detected by the absorbance at 230 nm. The purified amelogenins were characterized by SDS-PAGE, western blotting, the ALP-HPDL system and ELISA.

### Enzyme-linked immunosorbent assay (ELISA)

The identification of TGF-β1 and the amelogenin (P173, P162, P148 and P103)-TGF-β1 complexes *in vivo* was achieved by a sandwich enzyme immunoassay method using a Quantikine ELISA kit (R&D Systems, Inc., Minneapolis, MN, USA) (see “Enzyme-linked immunosorbent assay” in the Supplementary Methods).

### *in vitro* binding experiments between amelogenin and TGF-β1 or latent TGF-β1

The binding capacities of purified P173, P162, P148 and P103 amelogenins, TRAP and LRAP against the carrier-free recombinant human TGF-β1 (CF-hTGF-β1) (Cell Signaling Technology, Danvers, MA, USA) or the latent TGF-β1 were used for the *in vitro* binding experiments between amelogenin and TGF-β1 based on the method described in “*in vitro* binding experiments of amelogenin and TGF-β1” and “*in vitro* binding experiments of latent TGF-β1 and amelogenins” in the Supplementary Methods.

### Dynamic light scattering (DLS)

A custom-made multi-angle DLS instrument was used to measure the diffusion coefficient (*D*) of the amelogenin molecules. Because *D* calculated for each finite amelogenin concentration *c* was affected by the intermolecular interactions between the particles, the relationship between *D* and *c* was measured and extrapolated to the zero limit of *c* to estimate the actual diffusion coefficient, *D*_0_. The particle radius of the amelogenin molecules (*R*_H_) was calculated from *D*_0_ using the Stokes-Einstein relationship. The details of the equipment and calculation of *D* and *R*_H_ using the raw scattering data are described in the “DLS” section of the Supplementary Note 1.

The amelogenin solution was centrifugally filtered at 10,000 rpm using a 0.22-μm pore filter before each measurement, and the filtered solution was placed in a cylindrical glass cell with a 5-mm inner diameter (sample volume ~150 μL). The temperature was kept at 25.0 ± 0.1 °C using a thermostat-regulated water-circulating bath connected to the DLS instrument.

### Extraction of TGFBR1 in enamel organ epithelia and kinase assay

The TGFBR1 in enamel organ epithelia (approximately 0.5 g) corresponding to both secretory and transition stages was extracted with Mem-PER Plus Membrane Protein Extraction Kit (Thermo Scientific) and was characterized by SDS-PAGE and western blot analysis. The TGFBR1 kinase assay was performed using the LANCE Ultra TR-FRET (time-resolved fluorescence resonance energy transfer) technique (see “TGFBR1 kinase assay with TR-FRET” in Supplementary Methods).

### *in vitro* inactivation of TGF-β1 by MMP20 or KLK4

The CF-hTGF-β1 (1 μg) and the P103 amelogenin (10 μg)-TGF-β1 complex were dissolved in 100 μL of 50 mM Tris-HCl and digested with pMMP20 or pKLK4 (approximately 1 μg) for 20 h at 37 °C. Reaction aliquots at 0 and 20 h were characterized by SDS-PAGE and the ALP-HPDL system.

### Enzyme assay (ALP-HPDL system)

HPDLs were purchased from LONZA (LONZA, Walkersville, MD, USA). The cell culture and ALP activity assays were performed based on the method described in the “Enzyme assay” section of the Supplementary Methods.

### SDS-PAGE and Western blots

SDS-PAGE was performed using a Novex 16% Tris-Glycine gel (Life Technologies/Invitrogen, Carlsbad, CA, USA) and a 15% e-PAGEL mini gel (ATTO Corporation, Tokyo, Japan). The gel was stained with Simply Blue Safe Stain (Invitrogen) or ProteoSilver Stain (Sigma-Aldrich). The apparent molecular weights of the protein bands were estimated by comparison with the SeeBlue Plus2 Pre-Stained Standard (Life Technologies/Invitrogen) or the Novex Sharp Protein Standard (Life Technology/Invitrogen). Duplicate gels were transblotted onto Invitrolon polyvinylidene difluoride (PVDF) membranes (Life Technologies/Invitrogen) and immunostained with porcine amelogenin or TGFBR1 (Abcam, Cambridge, UK) polyclonal antibodies. Immunopositive bands were visualized by 3,3′-diaminodbenzidine (DAB) for amelogenin or enhanced chemiluminescence for TGFBR1. Full images of the blots that were cropped in the main figures are shown in Supplementary Fig. S12.

### Statistical analysis

For qPCR analysis and enzyme assays with the ALP-HPDL system, all values are represented as the means ± standard errors (s.e.m.). Statistical significance (*) was determined using an unpaired Student’s t-test. In all cases, p < 0.05 was regarded as statistically significant.

## Additional Information

**How to cite this article**: Kobayashi-Kinoshita, S. *et al*. TGF-β1 autocrine signalling and enamel matrix components. *Sci. Rep.* **6**, 33644; doi: 10.1038/srep33644 (2016).

## Supplementary Material

Supplementary Information

## Figures and Tables

**Table 1:** Amounts of the original or bound TGF-β1 for P173, P162, P148 and P103 amelogenins.

	(ng)	
Amelogenin	Original	Bound
P173	0.345 ± 0.037	1.176 ± 0.094
P162	0.201 ± 0.024	1.172 ± 0.136
P148	0.263 ± 0.096	1.062 ± 0.026
P103	0.797 ± 0.076	1.080 ± 0.066

The value indicates the total ng of TGF-β1 obtained from RP-HPLC before and after the *in vitro* binding experiments against 1 mg of protein.
