# Supplementary material for: TGF-β1 autocrine signalling and enamel matrix components
Source: Sci Rep. 2016 Sep 16;6:33644. doi: 10.1038/srep33644 (PMC5025654; doi:10.1038/srep33644)
Supplement: Supplementary Information [file srep33644-s1.pdf]

Supplementary Information

TGF-β1 autocrine signalling and enamel matrix components

Saeko Kobayashi-Kinoshita[1], Yasuo Yamakoshi[2*], Kazuo Onuma[3], Ryuji Yamamoto[2] and Yoshinobu Asada[1]

[1] Department of Pediatric Dentistry, School of Dental Medicine, Tsurumi University, 2-1-3 Tsurumi, Tsurumi-ku, Yokohama 230-8501, Japan.

[2] Department of Biochemistry and Molecular Biology, School of Dental Medicine, Tsurumi University, 2-1-3 Tsurumi, Tsurumi-ku, Yokohama 230-8501, Japan.

[3] National Institute of Advanced Industrial Science & Technology, Central 6, 1-1-1 Higashi, Tsukuba, 305-8566, Japan.

***Corresponding author:**
Yasuo Yamakoshi, PhD
Professor,
Department of Biochemistry and Molecular Biology
School of Dental Medicine, Tsurumi University
2-1-3 Tsurumi, Tsurumi-ku
Yokohama, 230-8501, JAPAN
Tel: +81-45-580-8374; Fax: +81-45-573-9599
E-mail: yamakoshi-y@tsurumi-u.ac.jp

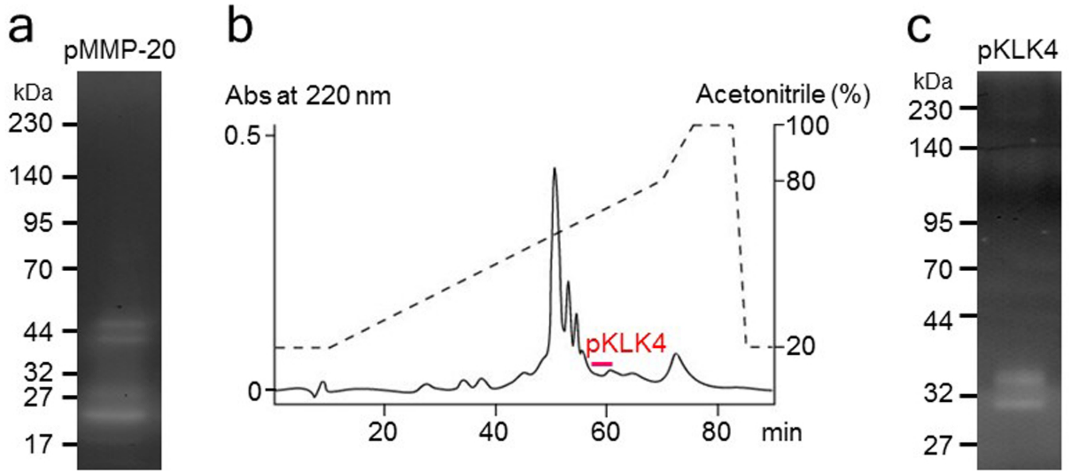

Supplementary Fig. S1. Isolation of porcine MMP-20 and KLK4. (a) Casein zymography showing final prepared MMP-20 obtained from the second eluate (S-AL-2) of heparin affinity chromatography for S-AL sample (see main Fig. 2b and 2c), **(b)** C18-chromatogram of H-N2 extracts, 40-65% ammonium sulfate precipitating and **(c)** gelatin zymography of KLK4 fraction collected at from 57.5 to 60 min (red line).

TGF-β1

```
pig   1:aldtnycfsstekncc vrqlyidfrkdlgwkwihepkgyhanfclgpcpyiwsldtqysk 60
```

```
pig   1:aldtnycfsstekncccvrqlyidfrkdlgwkwihepkgyhanfclgpcpyiwsldtqysk 60
```

TGF-β2

```
pig   1:aldaaycfrnvqdncclrplyidfkrdlgwkwihepkgynanfcagacpylwssdtqhsr 60
hum   1:aldaaycfrnvqdncclrplyidfkrdlgwkwihepkgynanfcagacpylwssdtqhsr 60
mus   1:aldaaycfrnvqdncclrplyidfkrdlgwkwihepkgynanfcagacpylwssdtqhtk 60
rat   1:aldaaycfrnvqdncclrplyidfkrdlgwkwihepkgynanfcagacpylwssdtqhtk 60

pig  61:vlslyntinpeasaspccvsqdlepltilyyigktpkieqlsnmivksckcs         112
hum  61:vlslyntinpeasaspccvsqdlepltilyyigktpkieqlsnmivksckcs         112
mus  61:vlslyntinpeasaspccvsqdlepltilyyigntpkieqlsnmivksckcs         112
rat  61:vlslyntinpeasaspccvsqdlepltilyyigntpkieqlsnmivksckcs         112
```

TGF-β3

```
pig   1:aldtnycfrnleenccvrplyidfrqdlgwkwvhepkgyyanfcsgpcpylrsadtthss 60
hum   1:aldtnycfrnleenccvrplyidfrqdlgwkwvhepkgyyanfcsgpcpylrsadtthst 60
mus   1:aldtnycfrnleenccvrplyidfrqdlgwkwvhepkgyyanfcsgpcpylrsadtthst 60
rat   1:aldtnycfrnleenccvrplyidfrqdlgwkwvhepkgyyanfcsgpcpylrssdtthst 60

pig  61:vlglyntlnpeasaspccvpqdlepltilyyvgrtakveqlsnmvvksckcs         112
hum  61:vlglyntlnpeasaspccvpqdlepltilyyvgrtpkveqlsnmvvksckcs         112
mus  61:vlglyntlnpeasaspccvpqdlepltilyyvgrtpkveqlsnmvvksckcs         112
rat  61:vlglyntlnpeasaspccvpqdlepltilyyvgrtpkveqlsnmvvksckcs         112
```

Supplementary Fig. S2. Alignment of the amino acid sequence of mature TGF-β1, TGF-β2 and TGF-β3 among pig, human, mouse and rat. The number of the last amino acid in each row is provided on the right. The red underline indicates a consensus amino acid sequence for heparin-binding.

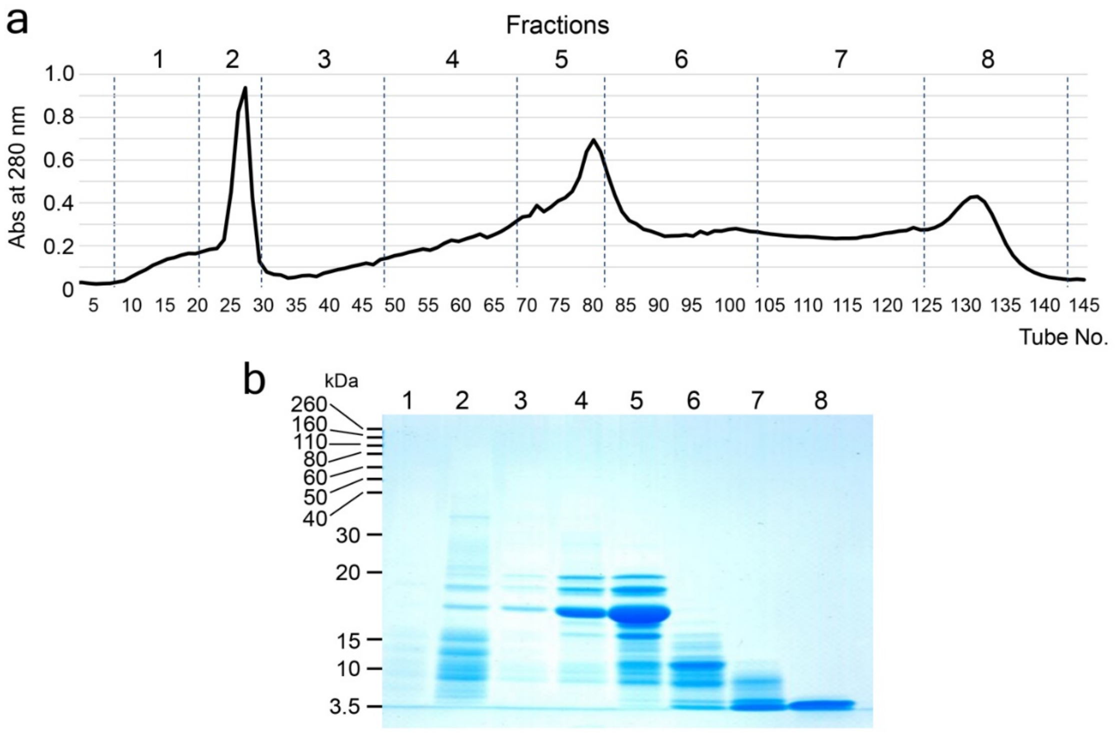

Supplementary Fig. S3. Fractionation of S-AL-1 extracts by size exclusion chromatography. (a) The chromatogram of the size exclusion fractionation of S-AL-1 extracts from Sephadex G-100 column and **(b)** the eight size exclusion fractions (peaks 1-8) separated on a 18% SDS-PAGE stained with Simply Blue.

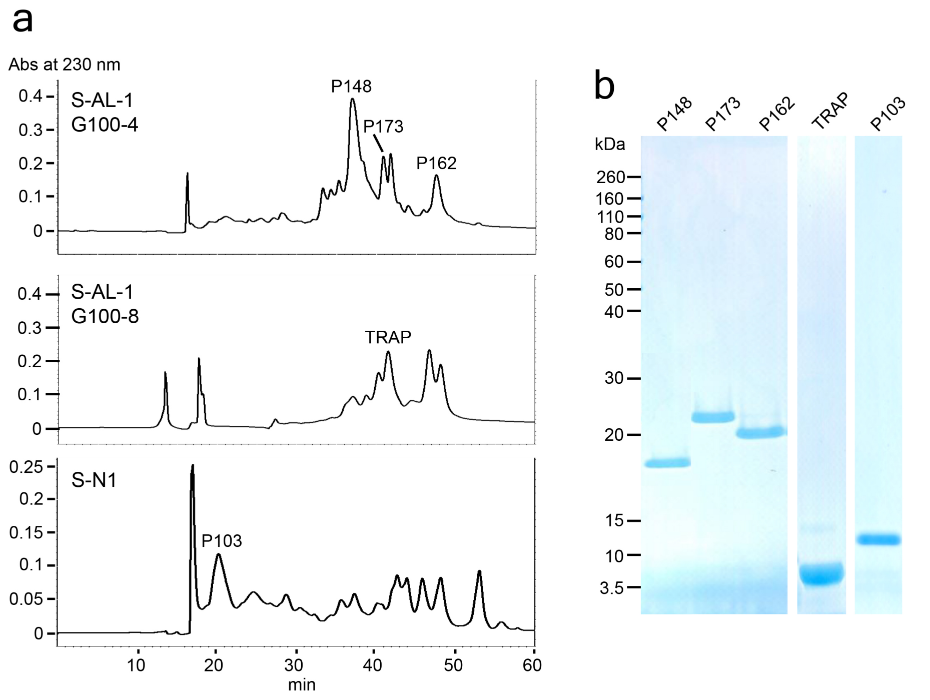

Supplementary Fig. S4. Isolation of major amelogenins by RP-HPLC. (a) Chromatogram of size exclusion fractions 4 (S-AL-1, G100-4) and 8 (S-AL-1, G100-8), and S-N1 fraction obtained from 40% saturation of ammonium sulfate fractionation for N extracts (see main Fig. 2a) further fractionated with a Discovery C18 column. **(b)** SDS-PAGE (15% gel) stained with Simply Blue showing isolated P173, P162, P148, P103 and TRAP amelogenins on RP-HPLC chromatograms.

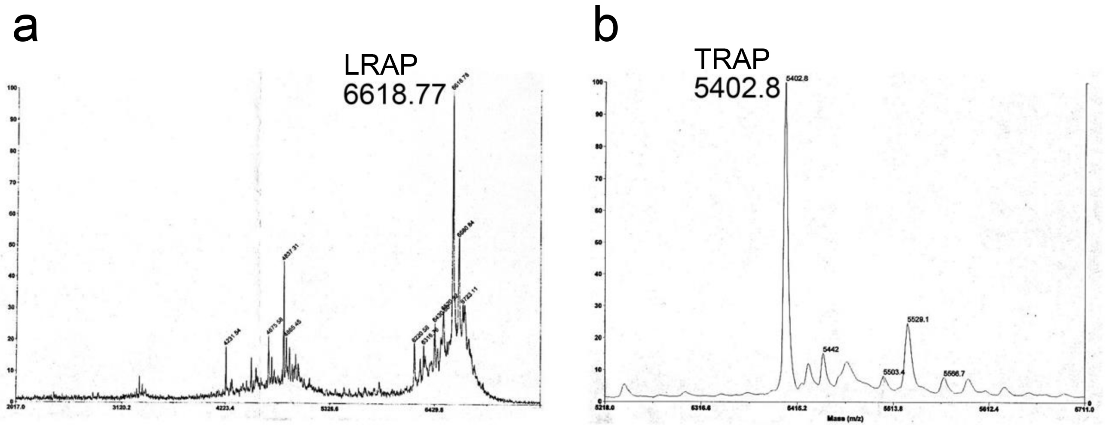

Supplementary Fig. S5. Mass spectrometry of LRAP and TRAP. (a) LRAP gave a value of 6618.77 Da (calculated mass, 6617.50), and **(b)** TRAP gave a value of 5402.8 (calculated mass, 5404.08), which closely matched the calculated masses of the single phosphorylated proteins.

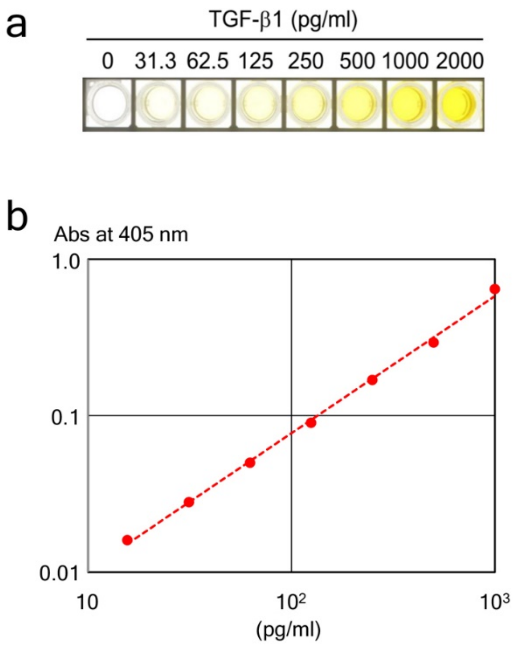

Supplementary Fig. S6. ELISA for the detection of TGF-β1. (a) Detection with tetramethylbenzidine substrate for serial dilutions of TGF-β1 ranging from 0 to 2000 pg mL^{-1} trapped to both TGF-β1 capture and detection antibodies. **(b)** Calibration curve between absorbance at 405 nm and concentrations of serial dilutions of TGF-β1.

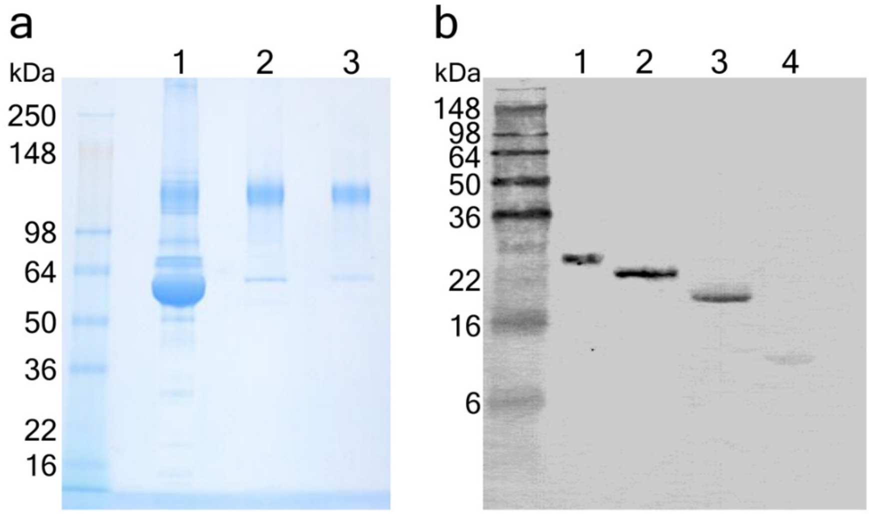

Supplementary Fig. S7. Preparation of biotinylated amelogenin antibody. (a) SDS-PAGE (5-20% gradient gel) stained with Simply Blue showing amelogenin antibody at each preparation steps. Lane 1: original amelogenin antibody, lane 2: purified amelogenin antibody after the removal of contaiminations by using IgG Purification Kit-A/G, lane 3: biotinylated amelogenin antibody prepared by using Biotin Labeling Kit-NH2. **(b)** Western blots (15% gel) used biotinylated amelogenin antibody showing each purified amelogenin. Lane 1: P173 amelogenin, lane 2: P162 amelogenin, lane 3: P148 amelogenin and lane 4: P103 amelogenin.

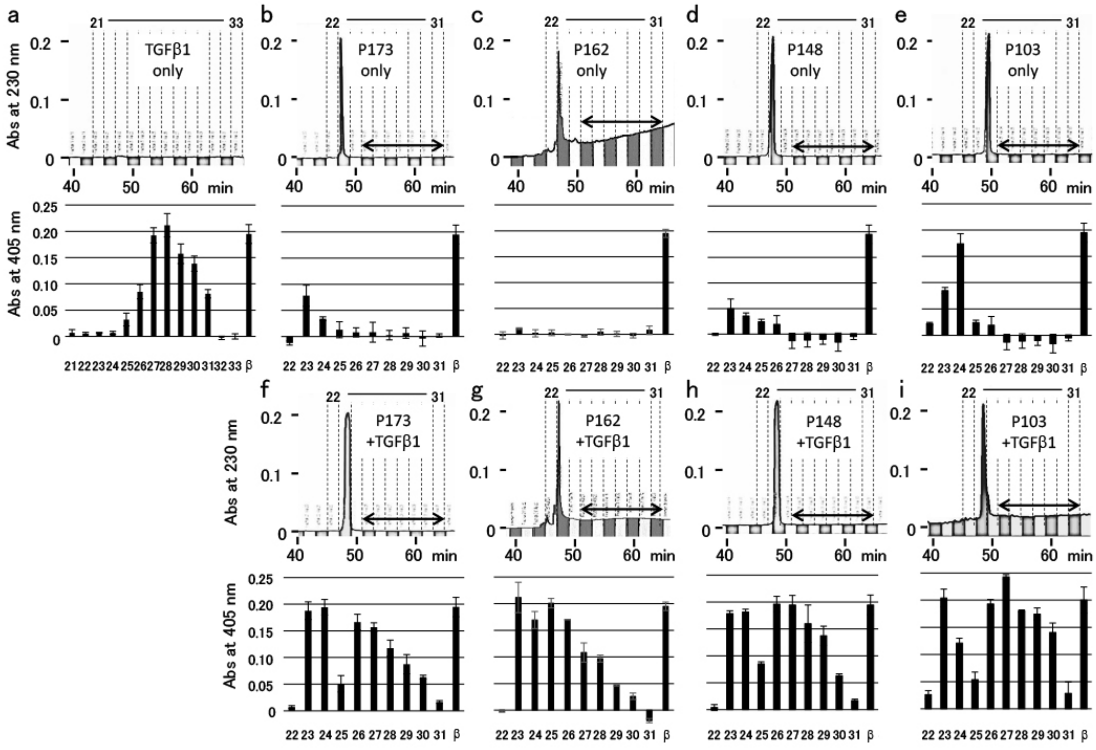

Supplementary Fig. S8. *In vitro* binding study of CF-hTGF-β1 for P173, P162, P148 and P103 amelogenins. (a-i) RP-HPLC chromatograms showing absorbance at 230 nm for all samples (top) and ALP-inducing activity of HPDL cells (bottom) exposed by fractions **(a)** 21-33 or **(b-i)** 22-31 in each samples. **(a)** CF-hTGF-β1 only. **(b-e)** Purified original amelogenin; **(b)** P173, **(c)** P162, **(d)** P148 and **(e)** P103 amelogenins. **(f-i)** TGF-β1-bound amelogenin; TGF-β1-bound **(f)** P173, **(g)** P162, **(h)** P148 and **(i)** P103 amelogenins. The recombinant human TGF-β1 with a carrier (0.3 ng mL^{-1}) (β) was used as positive control for the detection of ALP-inducing activity of HPDL cells (n = 9 culture wells for each sample).

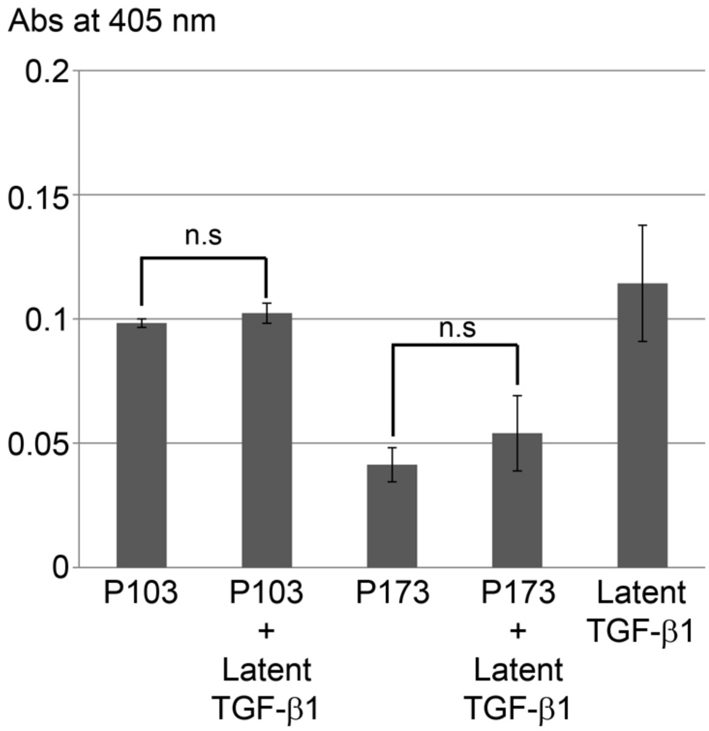

Supplementary Fig. S9. *In vitro* binding experiments of rh-latent TGF-β1 for P103 and P173 amelogenins. ALP-inducing activity of HPDL cells exposed by P103 amelogenin only, P103 amelogenin with latent TGF-β1, P173 amelogenin only, P173 amelogenin with latent TGF-β1 and latent TGF-β1 only samples (n = 9 culture wells for each sample).

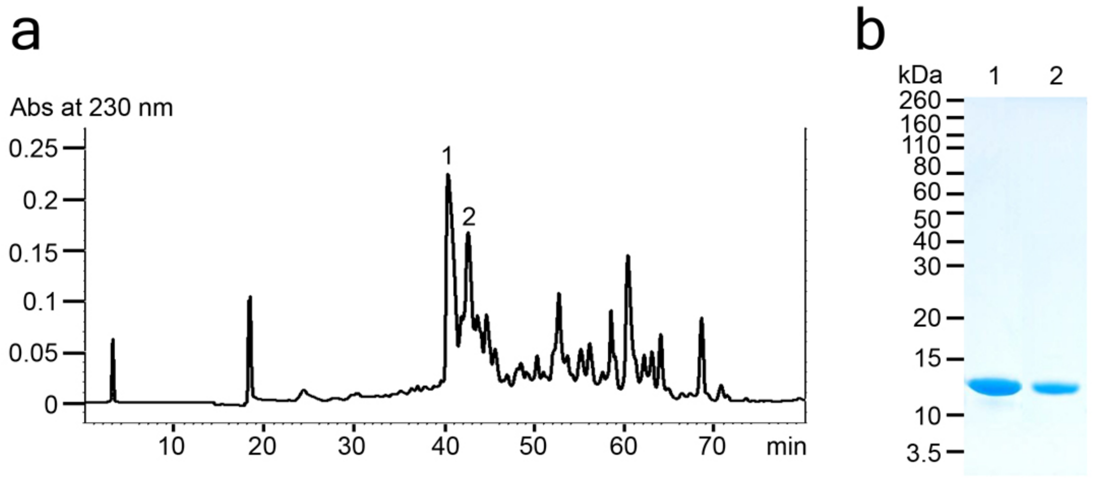

Supplementary Fig. S10. Purification of two TGF-β1-unbound P103 amelogenins. (a) RP-HPLC showing absorbance at 230 nm for the first eluate isolated from heparin affinity chromatography for S-N1 sample (see main Fig. 2f). **(b)** SDS-PAGE (15% gel) stained with Simply Blue showing peak 1 (P103-1 amelogenin) and peak 2 (P103-2 amelogenin) on RP-HPLC.

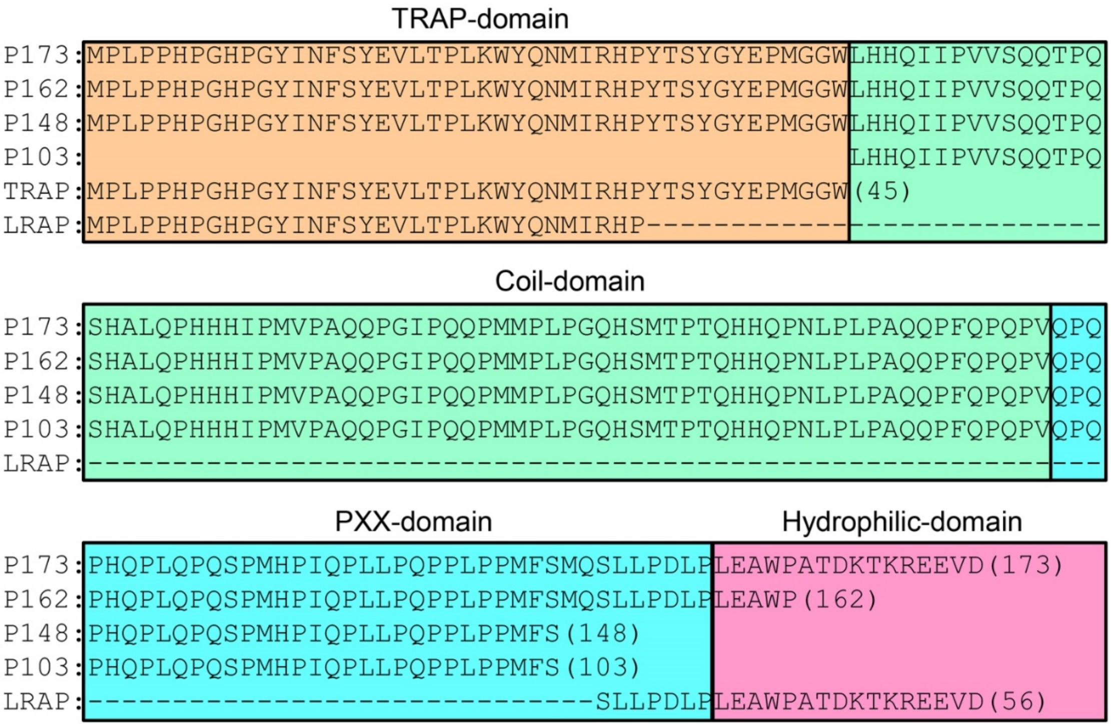

Supplementary Fig. S11. Amino acid sequences of porcine amelogenins. Numbers in parenthesis indicate the number of amino acid residues. A dotted line indicates the lacked sequence. Four functional domains; TRAP- (orange), Coil- (green), PXX- (blue) and hydrophilic-domains (pink) in porcine amelogenins are distinguished based on the mouse amelogenin sequence.

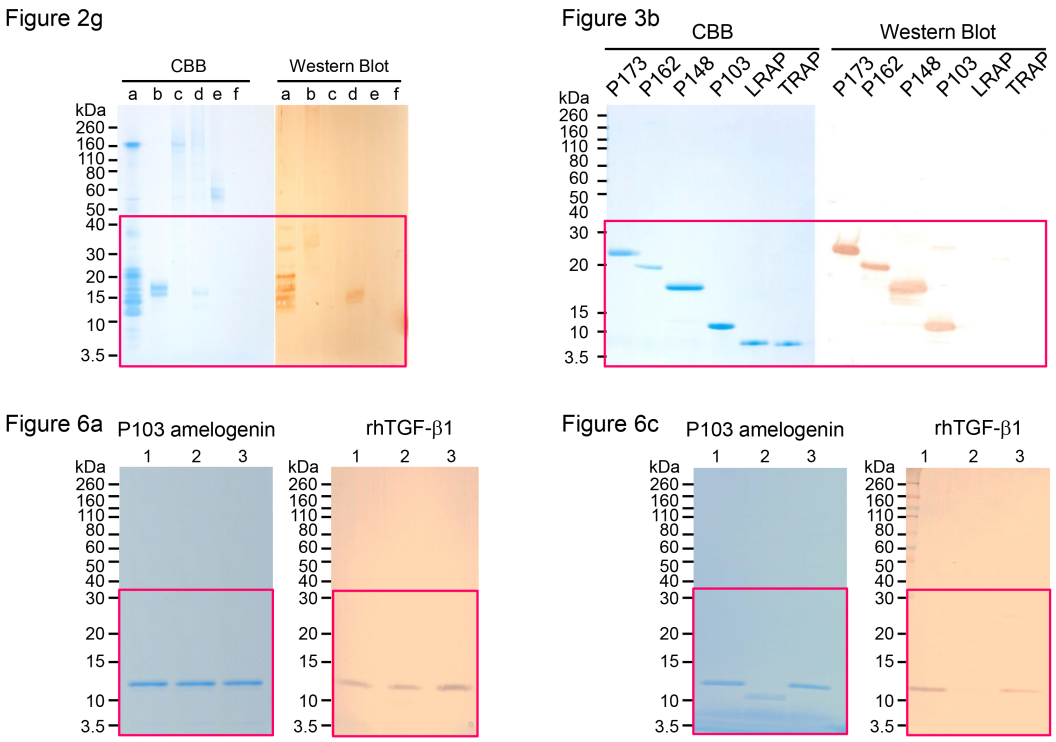

Supplementary Fig. S12. Uncropped images of all gels and Western blots shown in the main Figures. The cropped parts that are shown in the main Figures are marked by red boxes.

Supplementary Note

Dynamic light scattering (DLS)

In main Fig. 4, the light source of DLS was a solid-state laser with an output of 100 mW and a wavelength of 533 nm. The intensities at scattering angles, θ, from 30 to 100° with a step of 10° were simultaneously measured using eight optical fibers connected to eight photodiodes. The accumulation time to complete each measurement was 240 s. The second autocorrelation function, $g^{(2)}(\theta, t)$, at time t was measured for use in calculating the inverse decay time, Γ, of a particle:

$$g^{(2)}(\theta,t)-1=\left\{\sum K_i \exp(-\Gamma_i t)\right\}^2 (i=1,2,3), \tag{1}$$

where K is an instrumental constant and i represents the i-th particle in the solution. The measurement was repeated consecutively four times in each run to estimate the error in Γ. The $g^{(2)}(\theta, t)$ function was analyzed using an ALV-5000E correlation system. Both CONTIN and NNLS fittings were used to estimate Γ for each θ to determine whether the results were independent of the analysis method. In the calculation of Γ the intensity-percent and mass-percent fittings were compared to estimate the applicability.

The apparent translational particle diffusion coefficient, D, was calculated from the slope of the Γ vs. q^2 plot:

$$\Gamma = q^2 D, \tag{2}$$

where q is the scattering vector related to θ, and wavelength λ, and solution refractive index n:

$$q = 4\pi n \sin(\theta / 2) / \lambda . \quad (3)$$

The hydrodynamic radius (particle radius), R_H, was calculated using the Stokes-Einstein relationship:

$$R_H = \frac{k_B T}{6\pi\eta D_0}, \quad (4)$$

where k_B, T, and η are the Boltzmann constant, the absolute temperature of the solution, and the solution viscosity, respectively. The η was measured using a rolling-ball type viscometer (Lovis 2000M, Anton-Parr, Co. Ltd.)

Extraction of TGF-β in enamel matrix

Three TGF-β isoforms (TGF-β1 to -β3) have been identified in mammalian. Those isoforms possess over 97% of amino acid sequence homology within mature bioactive molecule[1]. Each of isoforms has also very high amino acid sequence among species (see Supplementary Fig. S2). Certain consensus sequences have been proposed for heparin-binding proteins, such as BBXB or BXBB, where B denotes a positively charged amino acid residue[2]. We found that the mature TGF-β1 possesses one heparin-binding site (R372-K375) conserved in mouse, rat, human and pig (see Supplementary Fig. S2), but not in the mature TGF-β2 or TGF-β3. Based on this finding, we tried to isolate TGF-β isoforms in both N1 and AL extracts with heparin affinity

chromatography.

Identification of amelogenin-TGF-β1 complex *in vivo*

The identification of TGF-β1 *in vivo* was determined by ELISA. The fractions (10 μg each) isolated by heparin column and each of purified P173, P162, P148 and P103 amelogenins (6.25 μg each) was dissolved or suspended in 200 μL of phosphate buffered saline (PBS), and aliquots (40 μL each) were used for ELISA. The quantitative analysis for TGF-β1 was carried out by using the calibration curve of serial dilutions of TGF-β1 ranging from 0 to 2000 pg mL^{-1} (see Supplementary Fig. S6).

In this study, we qualitatively modified a sandwich ELISA to know if amelogenin cleavage products bind to TGF-β1 *in vivo*. We first used the amelogenin antibody as the detection antibody as well as Western blot analysis, but the significant contamination (see Supplementary Fig. S7a: lane 1) caused the unspecific binding, that is, TGF-β1 only used as the negative control showed positive against this antibody. We therefore purified the antibody by using an IgG Purification Kit-A/G (Dojindo Laboratories, Kumamoto, Japan) (see Supplementary Fig. 7a: lane 2) and biotinylated with a Biotin Labeling Kit-NH2 (Dojindo) (see Supplementary Fig. 7a: lane 3). Western blot analysis showed that the purified P173, P162, P148 and P103 amelogenins reacted with the purified biotinylated-amelogenin and bound to a HRP-conjugated

streptoavidin for the DAB detection (see Supplementary Fig. 7b). We finally used this biotinylated-amelogenin antibody for detecting the amelogenin-TGF-β1 complex by ELISA (see main Fig. 3e).

Supplementary Method

Quantitative real-time PCR

Ameloblasts at three stages were extracted with RNA extraction reagent (Isogen, Nippon Gene Co., Ltd., Tokyo, Japan). Following the purified total RNA (2 μg) was reverse transcribed, the reaction mixture consisted of SYBR Green PCR master mix (Roche), supplemented with 0.5 μM forward and reverse primers and 2 μL of cDNA as template. After an initial preheating step at 95 °C for 10 min, samples were run for 45 cycles (denaturation for 10 sec at 95 °C, annealing for 10 sec at 60 °C and extension for 15 sec at 72 °C). The specific primer sets were designed using Primer-BLAST software (URL: http://www.ncbi.nlm.nih.gov/tools/primer-blast). The selected primers were designed as TGF-β1F, 5'-GCCTGCTGAGGCTCAAGTTA-3', paired with TGF-β1R, 5'- ATCAAAGGACAGCCACTCCG-3', MMP-20F, 5'-CTCGTCACGGCTCTGAAGTT-3' paired with MMP-20R 5'-CACCATGGAATTGCCTCCCT-3', KLK4-F, 5'-ATAAACGGCGAGGACTGCAA-3' paired with KLK4-R, 5'-GGGTTCTTGTTCGGGCTCAA-3' and TGFBR1-F,

5’-CCTAATTCCGCGAGACAGGC-3’, paired with TGFBR1-R, 5’-GCCAGATGGTGGCTTTCCTG-3’ to generate amplified products of 131 bp for TGF-β1, 171 bp for MMP-20, 183 bp for KLK4 and 145 bp for TGFBR1. GAPDH was used as the reference gene and selected primer was designed as GAPDH-F, 5’-CCATCACCATCTTCCAGGAG-3’, paired with GAPDH-R, 5’-ACAGTCTTCTGGGTGGCAGT-3’ to generate the amplified product of 346 bp. Each ratio was normalized the relative quantification data of TGF-β1, MMP-20, KLK4 and TGFBR1 in comparison to a reference gene (GAPDH) was generated on the basis of a mathematical model for relative quantification in qPCR system. All values were represented as means ± standard error (s.e.m.). Statistical significance (*) was determined using an unpaired Student’s t-test. In all cases, $p<0.05$ was regarded as statistically significant. The resulting data of MMP-20, KLK4 and TGF-β1 is shown in main Fig. 1b-d, while that of TGFBR1 is shown in main Fig. 5a.

Isolation of porcine kallikrein 4 (pKLK4)[3]

The H-N2 extracts was fractionated by reversed-phase high-performance liquid chromatography (RP-HPLC) using a Discovery C18 column (4.6 mm x 25 cm, Sigma‐Aldrich/Supelco, Bellefonte, PA, USA) and eluted with a linear gradient (20 to 80% B in 60 min) at a flow rate of 1.0 mL min^{-1} [3]. Buffer A was 0.05% trifluoroacetic acid (TFA); buffer B was 0.1% TFA in 80%

aqueous acetonitrile. Protein was detected by absorbance at 220 nm. The pKLK4 fraction eluted between 57 and 61 min, and was concentrated and buffer-exchanged to 50 mM Tris–HCl (pH 7.4) using an Amicon Ultra-4 Centrifugal Filter (Millipore Corporation). The KLK4 fraction was characterized by gelatin zymography (see Supplementary Method 3) and the protein content of the KLK4 fraction was measured using the Pierce 660 nm Protein Assay kit (Thermo Scientific, Rockford, IL, USA) and the sample was stored at -80 °C.

Enzymograms

To know the active form of purified pMMP-20 and pKLK4, zymography was carried out using Novex 12% Zymogram Casein Gel for pMMP-20 and Novex 10% Zymogram Gelatin Gel for pKLK4 (Life Technology/Invitrogen). Samples were dissolved in NuPAGE LDS sample buffer (Life Technology/Invitrogen), and electrophoresis was carried out at 30 mA for about 1 h with Novex Tris Glycine SDS running buffer (Life Technology/Invitrogen). The gel was shaken gently in 2.5% Triton X-100 solution for 1 h at room temperature with one buffer change and then incubated overnight with 10 mM $CaCl_2$ for pMMP-20 or 10 mM EDTA for pKLK4 in 50 mM Tris–HCl (pH 7.4) Proteinase activities were visualized as unstained bands after the gel was stained with Coomassie Brilliant Blue (CBB). The apparent molecular weights of the protein bands were estimated by comparison with DynaMarker Protein MultiColor III (BioDynamics

Laboratory Inc, Tokyo, Japan). The resulting zymograms are shown in Supplementary Fig. S1.

Enzyme-linked immunosorbent assay (ELISA)

For ELISA, the fractions (10 μg each) isolated by heparin column and each of purified P173, P162, P148 and P103 amelogenins (6.25 μg each) was bound to TGF-β1 capture antibody coated on the plate and was labeled by HRP-conjugated TGF-β1 detection antibody. The quantitative analysis of TGF-β1 was carried out based upon a calibration curve prepared from different concentrations of standard TGF-β1 (see Supplementary Fig. S6) Each purified P173, P162, P148 and P103 amelogenin bound to TGF-β1 capture antibody was labeled by the biotinylated amelogenin antibody (see Supplementary Fig. S7) as the detection antibody and HRP-conjugated streptavidin was subsequently added. The positive signal for TGF-β1 or amelogenin-TGF-β1 complex was detected using a tetramethylbenzidine (TMB) substrate.

***In vitro* binding experiments for amelogenin and TGF-β1**

Purified P173, P162, P148 and P103 amelogenins, TRAP and LRAP (0.5 mg each) were incubated with one μg of the carrier-free recombinant human TGF-β1 (CF-hTGF-β1) (Cell Signaling Technology, Danvers, MA, USA) in 50 mM Tris-HCl buffer (pH 7.4) for 20 h at 37°C. Each amelogenin was fractionated by RP-HPLC with the same system as their purification.

Each fraction was lyophilized, dissolved into 200 μL of water, and aliquots (50 μL) were used for the ALP-HPDL system. Amelogenins only were also incubated and fractionated by RP-HPLC as controls. Total amounts (ng) of original or bound TGF-β1 in fractions obtained from RP-HPLC before and after *in vitro* binding experiments against one mg of P173, P162, P148 and P103 amelogenins were calculated from the standard CF-hTGF-β1 (0.3 ng mL^{-1}). The resulting data is shown in Supplementary Fig. S8 and main Table 1.

***In vitro* binding experiments of latent TGF-β1 and amelogenins**

Purified P173 and P103 amelogenins (1 mg each) were incubated with 0.75 ng of rh-latent TGF-β1 in 50 mM Tris-HCl and 10 mM $CaCl_2$ (pH 7.4) for 20 h at 37°C. The P173 and P103 amelogenins bound or unbound to rh-latent TGF-β1 were fractionated by RP-HPLC and lyophilized. Each of samples was dissolved into 200 μL of water and an aliquot (50 μL) was used for the ALP-HPDL system. Amelogenins only were also incubated and fractionated by RP-HPLC as controls. The resulting data is shown in Supplementary Fig. S9.

TGFBR1 kinase assay with TR-FRET

Three experiments for TGFBR1 kinase assay were performed by using a technique of LANCE

Ultra TR-FRET. For ATP titration experiment, the TGFBR1 (10 μg) was incubated with 100 nM of ULight-Topo IIa (Thr1342) peptide as the substrate (PerkinElmer, Inc., Waltham, MA, USA) and serial dilutions of ATP ranging from 1 μM to 10 mM for 20 h at 37°C in kinase buffer (50 mM HEPES (pH 7.5) containing 1 mM EGTA, 10 mM $MgCl_2$ and 0.01% Tween-20). For the experiment of enzymatic time course, TGFBR1 was incubated with substrate and 5 mM ATP under the addition of 0.5 μg of CF-hTGF-β1 or 0.1 mg of P103 amelogenin-TGF-β1 complex for 1.5, 3, 6 and 12 h at 37°C in kinase buffer. For the experiment of enzyme inhibition curve, the TGFBR1 was incubated with the substrate, 5 mM ATP and serial dilutions of SB431542 ranging from 10 nM to 1 mM for 20 h at 37°C in kinase buffer. In all cases of experiments, the kinase reactions were terminated by the addition of 10 mM EDTA and incubated with 2 nM Europium (Eu)-labeled anti-phospho-DNA topoisomerase 2-alpha (Thr1342) antibody (PerkinElmer) for 1 h at room temperature. The intensity of the light emission corresponding to the level of substrate phosphorylation was detected with Varioskan LUX Multimode Microplate Reader in TR-FRET mode (excitation at 340 nm and emission at 665 nm) (Thermo Scientific). The resulting data is shown in main Fig. 5.

Enzyme assay (ALP-HPDL system)[4]

Human periodontal ligament fibroblasts (HPDL) were purchased from LONZA (LONZA,

Walkersville, MD, USA). The HPDL cells were distributed in 96-well plates at a density of approximately 5 x 10^5 cells/well and incubated for 24 hours. The growth medium was changed to contain with or without 10 nM 1α,25-dihydroxyvitamin D_3 and 10 μg mL^{-1} of samples dissolved in ultrapure water. After 72 additional hours of incubation, the cells were washed once with phosphate buffered saline (PBS), and ALP activity was assayed using 10 mM p-nitrophenylphosphate as the substrate in 100 mM 2-amino-2-methyl-1,3-propanediol-HCl buffer (pH 10.0) containing 5 mM $MgCl_2$ and incubated for 10 minutes at 37°C. Adding NaOH quenched the reaction, and the absorbance at 405 nm was read on a plate reader. Positive controls included the use of recombinant human TGF-β1 (rhTGF-β1) with carrier (0.3 ng mL^{-1}) (R&D Systems). The TGF-β1 receptor inhibitor, SB431542, was applied to a final concentration of 1 mM into the ALP-HPDL system for examination of the influence against the ALP-inducing activity increased by the application of samples. In controls, the ALP-inducing activity in HPDL cells was enhanced by rhTGF-β1.

Supplementary references

1. Lyon, M., Rushton, G. & Gallagher, J.T. The interaction of the transforming growth factor-βs with heparin/heparan sulfate is isoform-specific. *J Biol Chem* **272**, 18000-18006 (1997).

2. Shimazaki, K. et al. Approach to identification and comparison of the heparin-interacting sites of lactoferrin using synthetic peptides. *Excerpta Medica Int*

Congr Ser **1195**, 37-46 (2000).

3. Yamakoshi, Y. et al. MMP20 and KLK4 activation and inactivation interactions in vitro. *Arch Oral Biol* **58**, 1569-1577 (2013).

4. Nagano, T. et al. Porcine enamel protein fractions contain transforming growth factor-β1. *J Periodontol* **77**, 1688-1694 (2006).
